# Beta-boswellic acid facilitates diabetic wound healing by targeting STAT3 and inhibiting ferroptosis in fibroblasts

**DOI:** 10.3389/fphar.2025.1578625

**Published:** 2025-04-30

**Authors:** Ziyang Han, Weiping Wu, Zeming Bai, Yiping Xiu, Dapeng Zhou

**Affiliations:** Burn and Plastic Surgery Department, General Hospital of Northern Theater Command, Shenyang, China

**Keywords:** boswellic acid, STAT3, Ferroptosis, network analysis, diabetic wounds

## Abstract

**Objective:**

Diabetic wounds are a severe complication of diabetes, with persistently high incidence and mortality rates, often leading to severe clinical outcomes such as amputation. Beta-boswellic acid (β-BA) is a plant-derived pentacyclic triterpene with activities of inflammatory control and ferroptosis regulation. However, the protective effect of β-BA on DW has not been described.

**Method:**

We employed network analysis approaches and molecular docking to predict the potential targets and pathways of β-BA in the treatment of diabetic wounds (DW). Both *in vitro* and *in vivo* models were established, including high-glucose-induced fibroblast models and diabetic rat wound models. The effects of β-BA on diabetic wounds were investigated through CCK-8 assay, wound healing assay, immunofluorescence staining, western blotting, fluorescent probe analysis, gross observation, and histopathological experiments.

**Result:**

In this study, we predicted potential targets for β-BA using public databases and identified 29 key genes, with STAT3 being the most significant. GO analysis revealed that these targets are involved in biological processes closely related to ferroptosis, such as regulation of inflammatory response and lipid metabolism. Our results showed that HG induced ferroptosis in HSFs, as evidenced by decreased cell viability, altered GSH/MDA, Fe2+, and ROS levels, and changes in the expression of ferroptosis-related genes ACSL4 and GPX4. Notably, treatment with the ferroptosis inhibitor Ferr-1 partly reversed these effects. CCK-8 assays showed that β-BA improved HSFs viability in a concentration-dependent manner. Immunofluoresc-ence staining and further biochemical analyses demonstrated that β-BA reduced Fe2+ and lipid peroxide levels, prevented oxidative damage, and improved cell migration ability impaired by HG. Western blot analysis confirmed that β-BA reversed the changes in ACSL4 and GPX4 expression induced by HG. Molecular docking validated the potential binding between β-BA and STAT3. Western blot analysis revealed that β-BA increased the level of phosphorylated STAT3 in HSFs. Introducing a STAT3 inhibitor diminished the beneficial effects of β-BA on HG-induced cell dysfunction and suppressed its protective effect against ferroptosis. Finally, we assessed the efficacy of β-BA in the treatment of diabetic wounds in rats. BA administration accelerated wound closure, reduced inflammatory cell infiltration, improved granulation tissue arrangement, and increased collagen deposition. Immunohistochemical staining showed that BA upregulated the number of STAT3-positive cells and upregulated the number of GPX4-positive cells in the wounds, suggesting that BA can inhibit ferroptosis and accelerate wound healing in diabetic rats.

**Conclusion:**

Our findings suggested that β-BA may exert its therapeutic effects on diabetic wounds by targeting STAT3 and inhibiting ferroptosis.

## 1 Introduction

Diabetic wound (DW) is a worldwide issue that profoundly affects global health (Armstrong et al., 2023). A study in 2017 depicted that global prevalence of diabetic wounds has reached 6.3%. As the most serious complication of Diabetes, DW has resulted in lower-limb amputations with high morbidity and mortality, along with high cost of treatment (Ahmed et al., 2022; Walker et al., 2023). Despite endeavor aimed at elucidating the pathophysiological mechanisms of DW and developing new wound management strategies, the specific mechanisms remain to be clarified.

The healing process of diabetic wounds is significantly different from that of normal wounds (Sawaya et al., 2020). Chronic high glucose levels significantly impede wound healing by leading to the accumulation of reactive oxygen species in the mitochondria, which subsequently elevates oxidative stress and lipid peroxidation, causing fibroblast dysfunction and death (Khanna et al., 2010; Cheng et al., 2018). At the same time, disordered iron metabolism in DW leads to a high concentration of free ferrous ions in cells, further inducing ferroptosis (Liang et al., 2022; Sun et al., 2022).

Ferroptosis is a recently discovered programmed cell death (PCD) form that take place because of disordered lipid peroxidation and ferrous iron ions metabolism (Dixon et al., 2012). Studies have reported that ferroptosis in diabetic wounds hinders wound healing, forms a vicious circle of cell death with lipid peroxidation and excessive inflammatory reactions, which ultimately affects wound healing (Li et al., 2021; Feng et al., 2022). Wei et al. discovered that the inhibition of ferroptosis could activated PI3K/AKT signaling in diabetic wounds, resulting improvement in wound healing. Chen et al. reported better inflammatory control and weakened ferroptosis in HG-induced HUVECs after platelet rich plasma administration. In short, targeting ferroptosis could be a promising approach against diabetic wounds.

Beta-Boswellic acid (β-BA) was a mainly active pentacyclic triterpene metabolite derived from the gum of *Boswellia serrata (Roxb. ex Colebr,* Burseraceae*)*. According to traditional Chinese medicine (TCM) theory, symptomatic treatment of *Frankincense* has been broadly utilized to activated circulation and facilitate chronic wound healing for over 200 years. Modern research has reported β-BA as an anti-ferroptosis metabolite and its multiple targets. β-BA could regulate inflammatory in microglia and ferroptosis in astrocytes to facilitate ischemic stroke management (Liu et al., 2023); S670, an amid derivative of β-BA was reported to induce the ferroptosis of GBM. Despite studies have investigated its multiple targets, β-BA’s role in modulating oxidative stress and ferroptosis in diabetic wound remains unexplored.

This study initially focused on assessing the role of β-BA in diabetic wound healing. The *in vitro* experiments indicated that a High Glucose (HG) setting caused ferroptosis in fibroblasts. In fibroblasts, β-BA partially reversed lipid peroxidation and ferroptosis caused by HG, and a comparable effect was noted in the partial reversal of HG-induced STAT3 stimulation. An *in vivo* study in rat showed that β-BA partly reversed STZ-induced oxidative stress and ferroptosis in wound tissues. Furthermore, β-BA administration accelerated wound healing in diabetic rats. We conclude that β-BA facilitates diabetic wound healing by modulating HG-induced ferroptosis targeting STAT3.

## 2 Materials and methods

### 2.1 Materials and reagents

Human Skin Fibroblasts (HSFs) were purchased from American Type Cluture Collection (ATCC, USA). Cells were cultivated in Dulbecco’s modified Eagles medium (DMEM) at an environment of 37°C and 5% CO_2_. Medium was switched every 48 h. β-Boswellic acid (CAS: 631-69-6, purity ≥99.89%) was sourced from MedChemExpress (MCE, No. HY-N2513), derived from *Boswellia serrata* Roxb. ex Colebr. (Burseraceae; LSID:127067-1), Erastin (HY-15763), and Ferr-1(HY-100579) were obtained by MedChem-Express (MCE). The antibodies used were sourced from Abcam (Cambridge, UK): ACSL4 (ab155282), GPX4 (ab252833), STAT3 (ab68153), p-STAT3 (ab76315), VEGF (ab315238), β-actin (ab8226). In addition, iron assay kit, GSH, MDA, DAPI, DCFH-DA, was purchased from Beyotime (Shanghai, China).

### 2.2 Cell viability

Cell viability of HSFs was performed strictly abide the CCK8 Kit protocol (Beyotime, Shanghai). In a 96-well plate, HSFs were seeded at a density of 2 × 10^4^ cells per well. Afterward, they were treated with specified concentrations of Erastin, Ferr-1, and β-BA for 24 h, The plate was incubated at 37°C with 5% CO2 for a day. Afterward, 200 μl of fresh medium and 10 μl of CCK-8 solution were added to each well, followed by an additional 2-hour incubation, the absorbance was measured at a wavelength of 450 nm using a spectrophotometer for comparative analysis.

### 2.3 Fe2+ detection

To evaluate Fe^2+^ content in HSFs and tissues, vibrating homogenizer and ultrasonic cell disrupter were utilized to obtain supernatants. The quantification of iron levels was determined following the Iron Assay Kit instrument, and the final optial density was measured at 590 nm using a spectrophotometer for further analysis.

### 2.4 ROS determination

For intracellular ROS levels, cells on coverslips were treated with 10 μM DCFH-DA and incubated at 37°C for 30 min. After rinsing with PBS, cells were fixed, counterstained with DAPI, and analyzed using a confocal laser scanning microscope.

### 2.5 Quantification of GSH and MDA levels

Tissue and cell were homogenized and centrifugated as 2.3 described before, GSH and MDA activities were determined using the GSH Assay kit and MDA Assay kit. and following the instruction provided by the manufacturer.

### 2.6 Scratch wound assay

HSFs were cultured in 10%FBS/DMEM and seeded into six-well plates, 1 × 10^6^ cells were added to each well. 5 μg/ml mitomycin C solution was used to arrest cell mitosis. Following this, a 200 μl pipette tip was used to create scratches perpendicular to the plane of the 6-well plate. After washing three times with PBS, cells were subjected to Control group, HG group, and β-BA group. After 24 h culturing, the width of the scratches was observed under microscope and evaluated using ImageJ.

### 2.7 Western blot analysis

Cell total protein was extracted from HSFs treated with different interventions. Briefly, the protein samples were separated by sodium dodecyl sulfate-polyacrylamide gel electrophoresis (SDS-PAGE). The proteins were then transferred to polyvinylidene fluoride (PVDF) membranes, which were subsequently blocked with 5% skim milk. The membranes were incubated with primary antibodies overnight, followed by incubation with the corresponding horseradish peroxidase-conjugated secondary antibodies for 2 h. The blots were washed three times with tris buffered saline containing tween. Protein bands were detected using an electrochemiluminescence detection system, and their intensities were quantified using image analysis software.

### 2.8 Cell fluorescent staining

HSFs with different treatments were seeded into 6-well plates at a density of 1 × 10^5 cells per well. Following treatment with Dendrobine (10 and 40 μM) for 24 h, Subsequently, the cells were fixed with 4% paraformaldehyde for 10 min and permeabilized with 0.5% Triton X-100 at room temperature for 15 min. This was followed by blocking with 5% Bovine Serum Albumin (BSA) for 30 min, and then incubation with primary antibodies at 4°C overnight. After washing with PBS, the cells were incubated with appropriate fluorescent secondary antibodies conjugated with Cy3 for 1.5 h. They were then counterstained with DAPI for 5 min and imaged using a fluorescence microscope.

### 2.9 Histological analysis

The skin tissues were fixed overnight in a 4% paraformaldehyde solution and subsequently embedded in paraffin. They were then sectioned at a thickness of 4 μM. To assess collagen production, the sections underwent staining with both hematoxylin and eosin (H&E) and Masson’s trichrome stain (Beyotime). The microscopic examination was conducted using microscope, and photographs of the sections were taken.

### 2.10 Immunohistochemical staining

On the 14th day, the tissue slices were collected following the previously established procedure. Next, the slices underwent cooking and were then treated with a 10 mM sodium citrate solution at a pH of 6.0. Subsequently, the samples were washed and incubated with 10% fetal bovine serum (FBS) for 30 min. Thereafter, the tissue slices were stained with antibodies against CD31 at 1:200, and GPX4 at 1:200. Additionally, DAPI was applied to stain the nuclear DNA of the cells. Finally, fluorescent images were captured using a fluorescence microscope.

### 2.11 Network analysis

The conformation of β-Boswellic acid was downloaded from the Pubchem platform. SwissTargetPrediction, Drugbank, and BatMAN platforms were utilized to predict drug targets, then we Cross-verified the results obtained from both databases to ensure the reliability of the targets. Target of DW was obtained at OMIM and GeneCard database. Both were then imported into the Venny 2.1 platform to identify intersection targets between β-Boswellic acid and the disease. The intersection targets were imported into Cytoscape 3.11.2 to construct a network between β-Boswellic acid and DW targets. The intersection targets were imported into the STRING database for protein interaction analysis. The extracted TSV file was imported into Cytoscape’s built-in BisoGenet tool to generate the PPI network. CytoNCA was used to analyze the attribute values of each node in the network, calculate the topological characteristics of the PPI network, and screen for core targets, thereby identifying the core targets. At last, the core targets were imported into the Metascape platform for Gene Ontology (GO) functional enrichment analysis and Kyoto Encyclopedia of Genes and Genomes (KEGG) pathway enrichment analysis.

### 2.12 Molecular docking analysis

We first utilized ChemDraw to depict the structure and minimize the energy of β-BA, the 3D structures of the proteins were obtained from Universal Protein database. A blind docking strategy was adopted, and no specific binding pocket of proteins was predefined to comprehensively explore the potential binding modes between β-boswellic acid (β-BA) and STAT3 protein (PDB ID: 2OV5). The docking simulation was carried out using AutoDock Vina 1.2.1, parameters were selected the “default” parameter options. −5.0 kcal/mol was set as the binding energy threshold, and the conformations with binding energy lower than this value were regarded as potential binding modes. Finally, PYMOL was employed to create a three-dimensional (3D) visual representation of the results.

### 2.13 *In vivo* rat experiments

The animal protocol complies with NIH guidelines (Guide for the Care and Use of Laboratory Animals, No. 85–23, revised 1996), and was approved by Ethical Committee of General hospital of northern theater command (NO. 20230241).

#### 2.13.1 Protocol 1:The establishment of a diabetic rat model

Male Sprague–Dawley rats weighing 180–200 g were given a high-fat-sugar diet for a month, followed by an 8-hour fast from food and water, and then injected with 40 mg/kg of streptozotocin intraperitoneally. Following a 3-week period of continuous random blood glucose monitoring, rats with RBG levels over 11.1 mM were designated as the diabetes group, whereas the control group was injected with PBS.

#### 2.13.2 Protocol 2 creation of full-thickness skin loss models

Rats in protocol one received an intraperitoneal injection of 1% Pentobarbital sodium solution for anesthesia. A circular full-thickness skin incision with a 1 cm diameter was made on the rat’s dorsal midline using a Sampler (Miltex, China), and the wound was then covered with sterile gauze.

#### 2.13.3 Protocol 3 drug administration and group

Rats participating in protocol two were given either PBS or β-BA. 10 mg/kg dose of β-BA was administered subcutaneously beginning on the second day post-creation of wound models. The rats were divided randomly into three groups: group 1 consisted of normal rats treated with PBS (Control); group 2 included diabetic rats treated with PBS (STZ); and group 3 comprised diabetic rats treated with β-BA.

## 3 Result

### 3.1 Prediction and analysis of potential targets for β-BA

**FIGURE 1:**
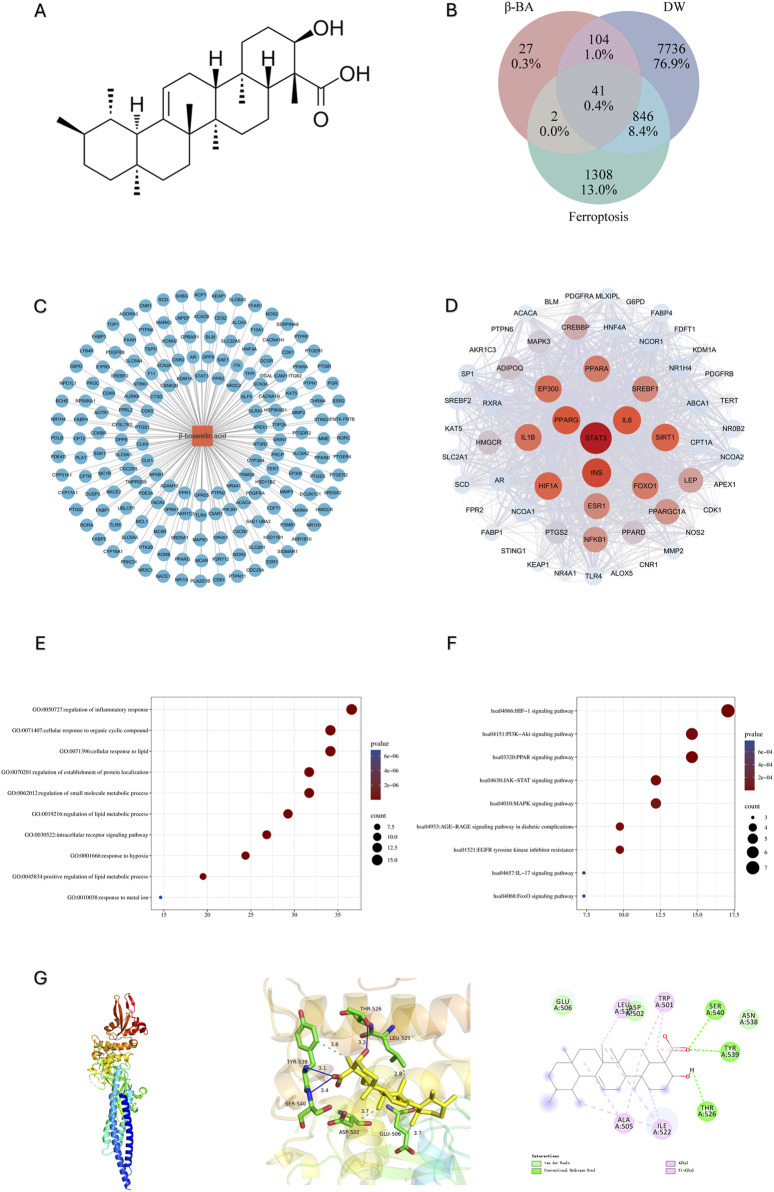
Prediction and analysis of potential targets for β-BA. **(A)** The molecular structure of β-BA; **(B)** The intersecting targets of DW, β-BA and ferroptosis; **(C)** Metabolite-target interaction of β-BA; **(D)** Major cluster extracted from the protein–protein interaction (PPI) network indicating main functional gene clusters of potential targets. **(E, F)**: The GO analysis and KEGG analysis reveals enriched biological process (BP) and enriched pathways in the treatment of diabetic wounds (DW). **(G)** molecular docking result between BA and STAT3.

To gain insight into the potential target and underlying mechanism, firstly we obtain β-BA’s molecular structure from Pubchem (Figure 1A), SwissTargetPrediction database reported a total of 174 potential targets for β-BA (Figure 1C). Then we retrieved 8727 disease targets related to DW from GeneCard and OMIM. A total of 2197 Targets of ferroptosis was obtained from Ferdb. The intersection of them was shown with a Venn diagram (Figure 1B). The PPI network screening of intersection targets identified 29 key genes with significant structural and functional importance, among which STAT3 had the highest score (Figure 1D). It is noteworthy that GO analysis indicates that the core target is involved in several biological processes, including.

Regulation of inflammatory response; cellular response to lipid; regulation of lipid metabolism; and response to metal ions, which are closely related to ferroptosis (Figures 1E,F).

### 3.2 High-glucose environment induces ferroptosis in HSFs

**FIGURE 2:**
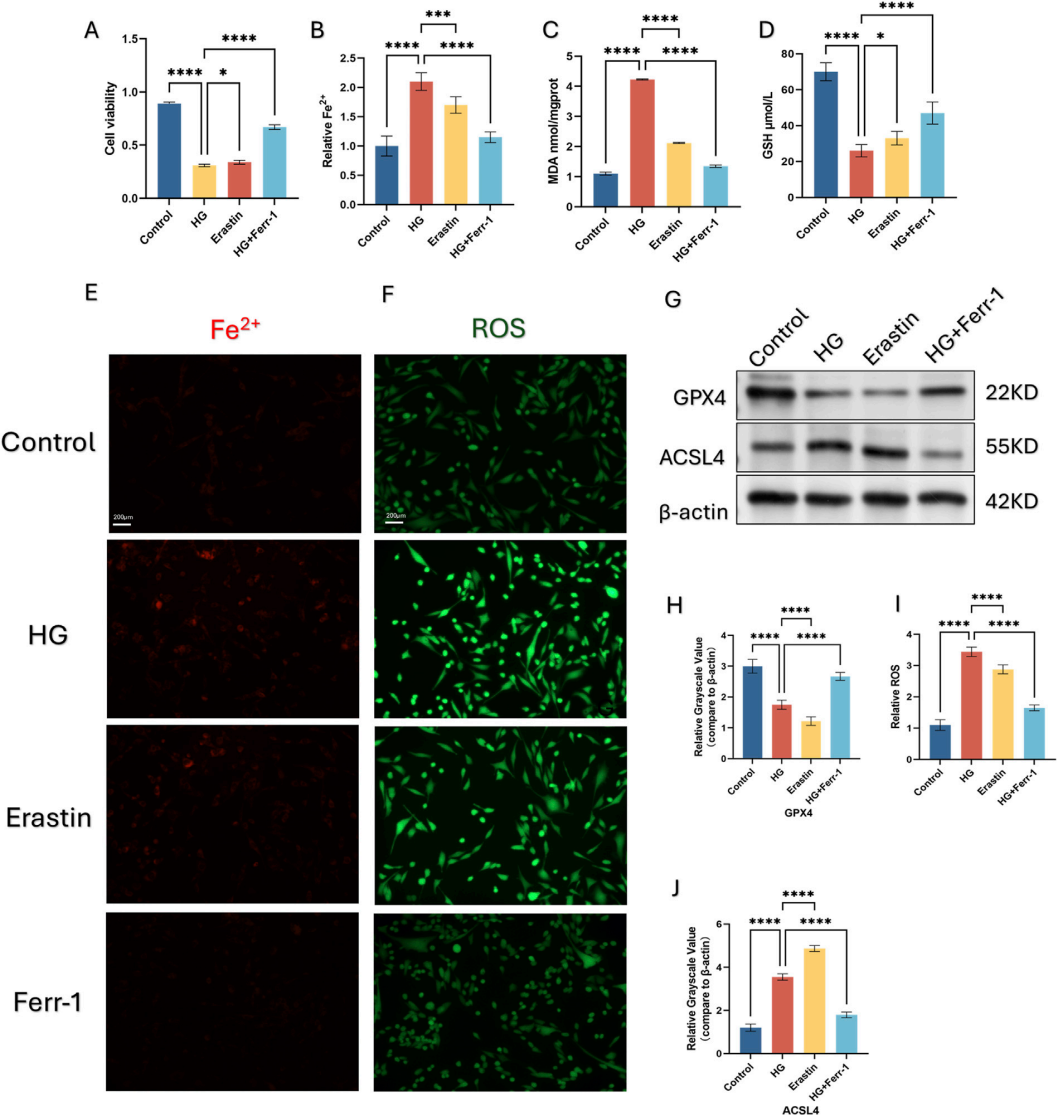
Ferroptosis is induced by high glucose (HG) in HSFs. **(A)** CCK-8 assay of HSFs was conducted to measure relative viability after being treated with HG, ERASTIN and Ferr-1. **(B)** measurement of Cell Ferrous iron assay detected by fluorometric. **(C, D)**: The content of GSH and MDA was quantified using a commercial kit. **(E, F)** Representive immunofluorescence images of Fe2+ and ROS, red fluorescence indicates Fe2+, green fluorescence indicates ROS. **(G, H, J)** the protein expression of the ferroptosis related gene GPX4 and ACSL4 under different treatment using Western blot (WB), *p < 0.05, **p < 0.01, **p < 0.001, ****p < 0.0001 **(I)** measurement of ROS using DCFH-DA kit. All data are from three-time independent experiments.

To mimic the high-glucose microenvironment of HSFs *in vivo*, we cultured HSFs in medium at a dosage of 25.5 mM glucose (Yang J. Y. et al., 2024) for 48 h. HSFs were divided into Control, HG, Erastin, HG+Ferr-1. Cell viability was accessed by CCK-8 assays. Treatment of 25.5 mM HG and Erastin both decreased HSFs cell viability, while this trend was partly reversed by the inhibitor of ferroptosis (Figure 2A). Further detection of GSH, MDA, Fe^2+^, and ROS revealed that HG treatment could exert similar influence as Erastin on HSFs, while this influence could partly be rescued by the inhibition of Ferroptosis (Figures 2B–E, I). Moreover, HG exposure upregulated the expression of ACSL4 and downregulated the expression of GPX4, while treatment with Ferr-1 could partly reverse these trends (Figures 2G,H,J).

### 3.3 β-BA rescues cell viability caused by HG exposure in HSFs

**FIGURE 3:**
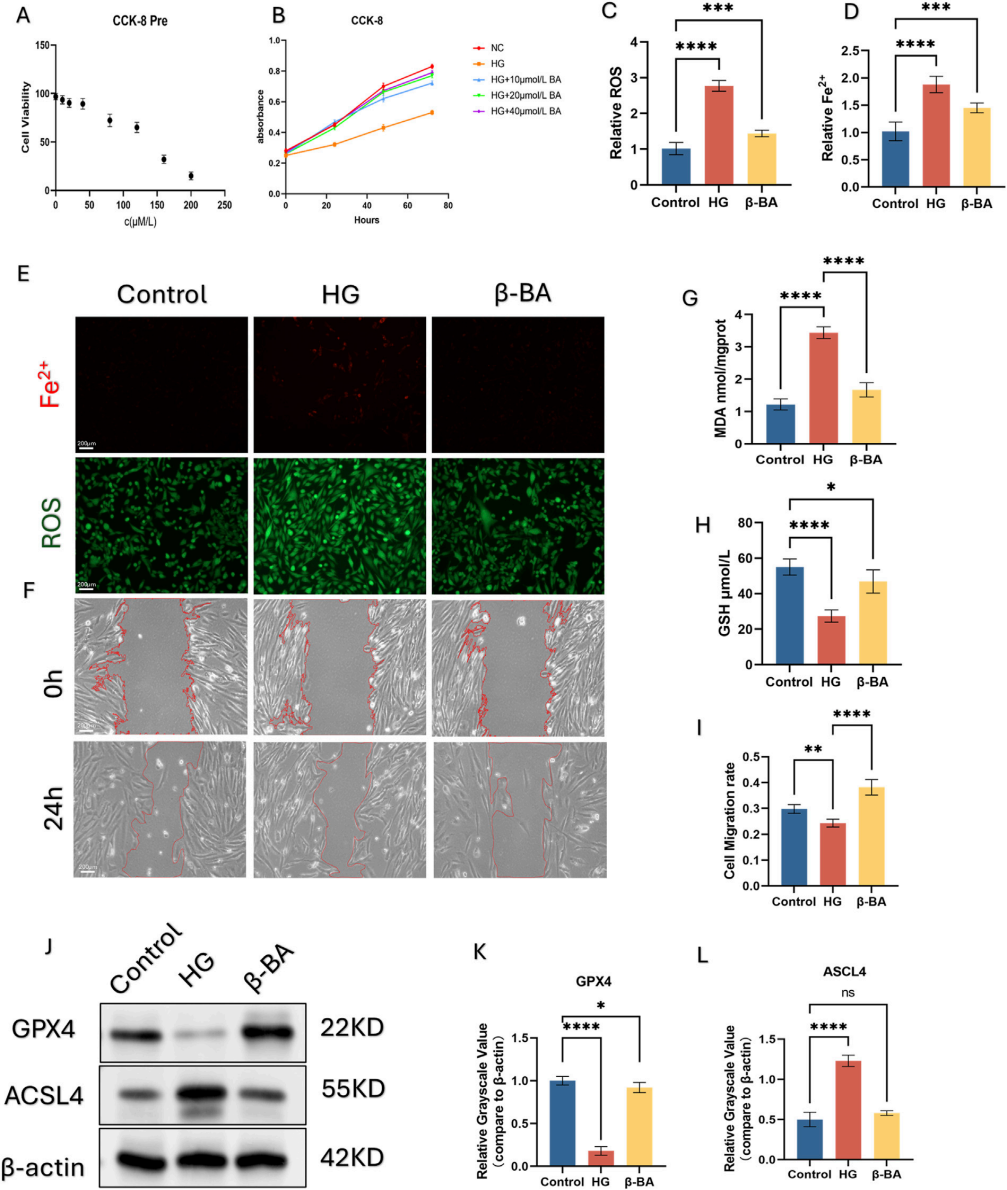
β-BA improve HSFs cell activity by alleviating ferroptosis. **(A, B)** Effective concentration of β-BA was investigated using CCK-8 assay. **(C, D)** Intracellular Fe2+ and ROS were detected by immunofluorescence probe. **(E)** Representive immunofluorescence images of Fe2+ and ROS, red fluorescence indicates Fe2+, green fluorescence indicates ROS. **(F)** Representative image of a cell wound healing assay, with the red line denoting the boundary of the scratch. **(G, H)** The content of GSH and MDA was quantified using a commercial kit. **(I)** The measurement results of the wound healing rate in the cell scratch assay. **(J–L)** the protein expression of the ferroptosis related gene GPX4 and ACSL4 under different treatments using Western blot (WB), *p < 0.05, **p < 0.01, ***p < 0.001, ****p < 0.0001; All data are from three-time independent experiments.

CCK-8 assay revealed that the highest concentration of β-BA that was safe was 40 μM. What’s more, the administration of β-BA could significantly improve the decrease in HSFs viability induced by high glucose in a concentration-dependent manner (Figures 3A-B).

### 3.4 β-BA attenuates ferroptosis and protected cell function induced by HG in HSFs

Here we examined β-BA capacity to inhibit HG-induced ferroptosis. HSFs was divided into Control, HG, HG+β-BA groups. Immune Fluorescence staining (IF) of Fe2+ and intracellular lipid peroxides (ROS) were performed to investigate the protection of β-BA (Figures 3C, D, E). The fluorescence intensity of Fe2+ and lipid peroxides significantly rose under HG stimulation, while the administration of β-BA partly reversed this effect. Further detection of GSH/MDA demonstrated that β-BA prevented oxidative damage induced by HG (Figures 3G, H, E). Wound healing assays were performed to evaluate cell function. As depicted, Cell migration ability in the HG group was significantly lower than that in the Control group, β-BA can improve the impaired migration ability of HSFs under high glucose conditions in a concentration-dependent way (Figures 3F, I). Western blot analysis showed that the expression of ACSL4 was upregulated and that of GPX4 was downregulated under HG induction, while the posttreatment of β-BA reversed these changes (Figures 3J–L).

### 3.5 BA protected HSFs function against ferroptosis via the STAT3

**FIGURE 4:**
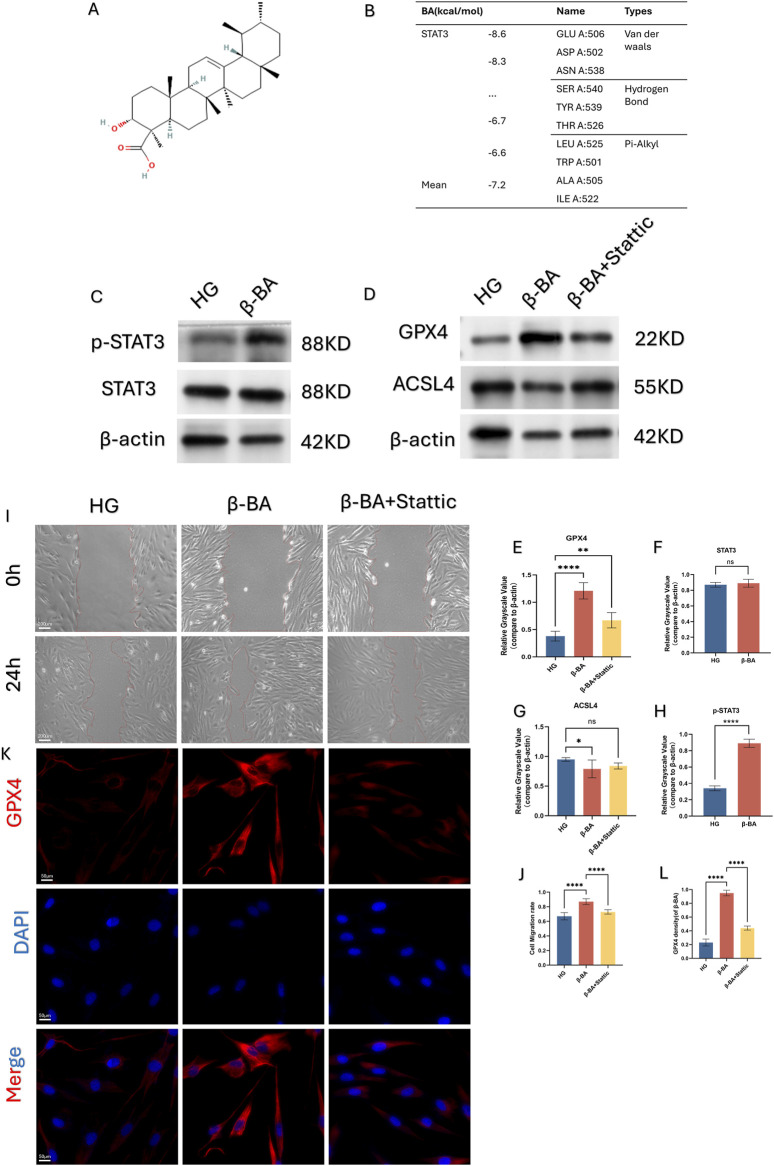
β-BA may regulate ferroptosis and cellular function by affecting the phosphorylation level of STAT3. **(A, B)** Molecular docking validates the binding site interaction between β-BA and STAT3. **(C–H)** the protein expression of Phosphorylated STAT3 (p-STAT3) and STAT3 under treatment of β-BA; the protein expression of the ferroptosis related gene GPX4 and ACSL4 under treatment of STAT pathway inhibitor, ^ns^p>0.05,*p < 0.05, **p < 0.01, ***p < 0.001,****p < 0.0001. **(I, J)** Representative image of a cell wound healing assay, with the red line denoting the boundary of the scratch under the treatment of Stattic. **(K, L)** The relative fluorescent expression level of GPX4 was determined through immunofluorescence experiments, where red fluorescence represents GPX4, blue fluorescence represents DAPI, and the mixed fluorescence represents the merge staining of both. All data are from three-time independent experiments.

Through network analysis, the potential biological target of β-BA was identified as STAT3, as illustrated in the figure. Subsequently, molecular docking was employed to validate the potential binding between β-BA and STAT3. The spatial structure of STAT3 was found to interact with β-BA via hydrogen bonds, with a binding energy of −8.6 (kcal/mol) (Figures 4A, B,1G). Western blot analysis revealed that BA administration increased the level of phosphorylated STAT3 in HSFs, However, the impact on STAT3 expression is negligible (Figure 4C, F, H). To further investigate, we introduced a STAT3 inhibitor (Stattic) into the experimental system and divided the cells into three groups: HG group, BA group, and BA + STAT3 inhibitor group. In subsequent cell function experiments, it was observed that the addition of the STAT3 inhibitor diminished the beneficial effects of BA on HG-induced cell dysfunction (Figures 4I, J). Furthermore, due to the action of the STAT3 inhibitor, the protective effect of BA against ferroptosis was also suppressed. As shown in the Figure (Figures 4D,E, G, K, L), the introduction of Stattic counteracts the improvement effect of β-BA on ferroptosis-related gene and significantly reduces the relative fluorescence intensity of the ferroptosis-related gene GPX4. In summary, inhibiting STAT3 abolished the beneficial effects of BA, suggesting that β-BA may exert its biological functions by targeting STAT3.

### 3.6 BA promotes wound healing and wound angiogenesis in diabetic rats

**FIGURE 5:**
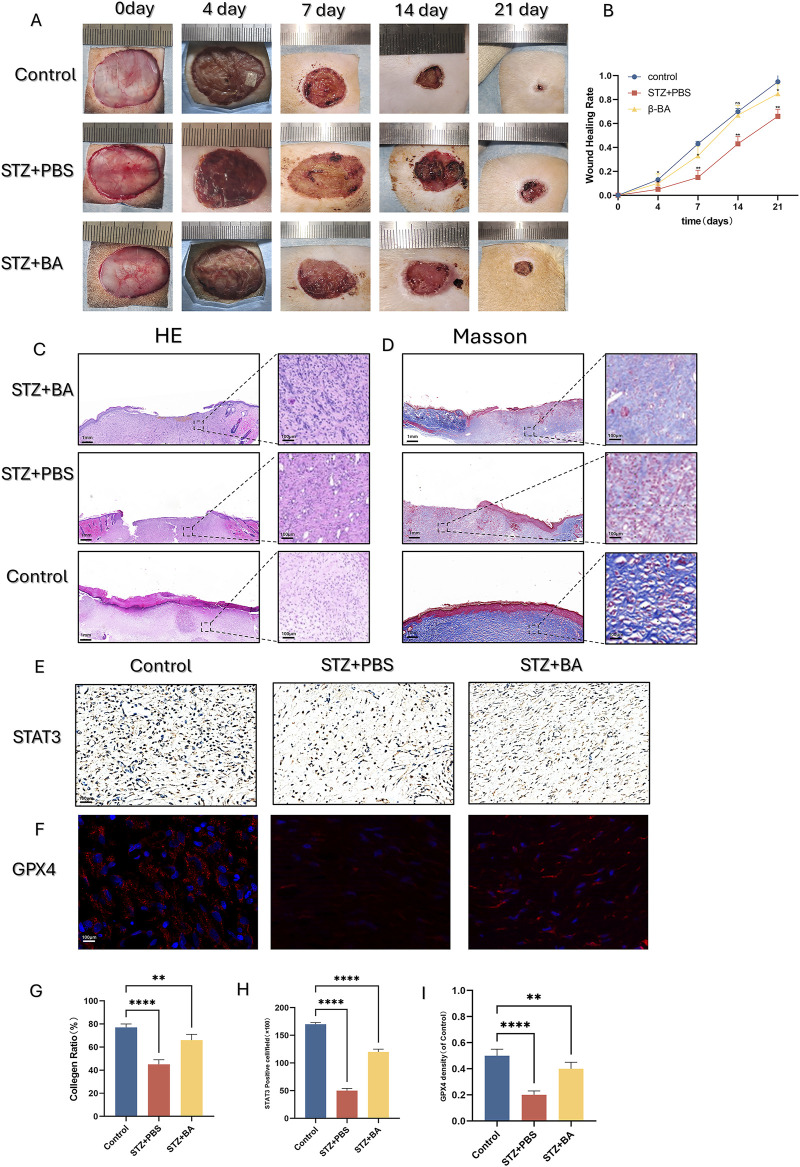
β-BA promotes diabetic wound healing process in STZ-induce rat. **(A, B)** Representative digital images of wound healing for control, PBS and β-BA administration group on day 0, day 4, day 7, day 14, day 21. **(C, D, G)** Representative images of H&E and Masson staining, as well as quantitative analysis of collagen ratio, this assay was performed on day 21 on full-thickness wounds. **(E, H)** Representative images and quantitative analysis of the STAT3 immunohistochemical staining were taken on day 14 after the operation. **(F, I)** representative images of immunofluorescence staining for GPX4 from each of the different groups, as well as quantitative analysis of the staining. *p < 0.05, **p < 0.01, ***p < 0.001, ***p < 0.0001; All data are from three-time independent experiments.

To assess the efficacy of BA in the treatment of diabetic wounds, we induced diabetes in rats through intraperitoneal injection of streptozotocin (STZ) and subsequently created full-thick skin wounds on their mid-back. The wound healing curve demonstrated that the wound healing rate was significantly reduced in the diabetic group, whereas BA administration accelerated wound closure, although this promotional effect is not as significant as that in the control group (Figures 5A, B).

We further observed HE staining and Masson staining on day 21: In the BA-treated group, partial wounds were epithelialized, with less inflammatory cell infiltration, denser granulation tissue arrangement, and more pronounced collagen deposition (Figures 5C,D,G). Immunohistochemical staining provided similar insights, showing that BA administration upregulated the number of STAT3-positive cells in the wounds (Figures 5E, H). Furthermore, BA also significantly increased the number of GPX4-positive cells in the wounds (Figures 5F, I). This finding suggests that BA can inhibit ferroptosis in diabetic wounds and accelerate wound healing.

## 4 Discussion

In this study, we utilized *in vitro* and *in vivo* diabetic models combined with bioinformation method to elucidate that β-BA reverses HG-induced ferroptosis also partly reverses HG-induced oxidative stress and STAT3 suppression in fibroblasts. Additionally, BA administration in rats ameliorate diabetes mediated ferroptosis as well as oxidative stress and angiogenesis in diabetic rats. Finally, we reported that β-BA significantly accelerates diabatic wound healing. These observations provide a new perspective to reveal the pathogenesis of wound healing and provide a new theoretical basis for applying β-BA to treat diabetic wounds.

DW is known for its complex pathological mechanisms and numerous therapeutic targets. Network pharmacology analysis facilitates precise and effective therapeutic interventions by skillfully transforming disease phenotypes into intrinsic phenotypic associations encompassing multiple targets and diseases (Nogales et al., 2022). Boswellic acid is a natural pentacyclic triterpenoid metabolite extracted from frankincense resin and was traditionally used to treat conditions like chronic inflammation in TCM theory. Various preclinical studies have demonstrated its enormous potential in the treatment of asthma, arthritis, cerebral edema, chronic intestinal diseases, chronic pain syndromes and cancers (Yadav et al., 2012; Zhou et al., 2015; Mazzio et al., 2017; Majeed et al., 2019). Excitingly, a RCT clinical studies have supported the effects of Boswellia serrata gum who showed significant reductions in fasting blood glucose, HbA1c, and serum insulin levels, as well as decreases in serum cholesterol, low-density lipoprotein (LDL), and triglycerides (Azadmehr et al., 2014). In this study, by utilizing network pharmacology analysis and molecular docking, we revealed a close relationship between β-BA and lipid oxidation as well as iron ion metabolism. Venne diagram showed that nearly 1/3 β-BA’s core targets were involved in either DW or Ferroptosis. GO and KEGG analysis of these core targets revealed their potential connection to lipid metabolism, ion metabolism, responses to inflammation and notably STAT3 differential activation. Additionally, we conducted molecular docking between β-BA and top ten core targets to provide the possibility for the binding of both. The abovementioned findings implied that β-BA might exert regulatory effects on DW by regulating inflammation-related signaling pathways. While our network analysis provided valuable insights into the potential targets of β-BA in diabetic wound healing, several limitations must be acknowledged: the predictive nature of network analysis relies on existing databases (e.g., SwissTargetPrediction, GeneCards), which may prioritize well-characterized targets and introduce selection bias, To address these limitations, we cross-validated key targets using ChEMBL and experimentally confirmed STAT3’s role through functional assays.

Based on available literature, Chronic exposure to hyperglycemic cause increased inflammation in the cellular environment of skin tissues, which in turn lead to intracellular oxidative stress and lipid peroxidation and finally results in ferroptosis (Shah et al., 2018; Dixon and Olzmann, 2024). Ferroptosis is a unique form of cell death, and its mechanism is closely related to ROS (reactive oxygen species), GSH (glutathione), and iron metabolism (Dixon et al., 2012). When the intracellular levels of lipid reactive oxygen species (L-ROS) exceed the antioxidant capacity of GPX4 (glutathione-dependent peroxidase), it leads to lipid peroxidation, which is one of the key features of ferroptosis (Stockwell et al., 2017). Meanwhile, GSH, as an important intracellular antioxidant, its depletion further exacerbates oxidative stress and promotes the occurrence of ferroptosis (Gao and Jiang, 2018). In addition, iron metabolism also plays a central role in ferroptosis, and iron overload can generate more ROS through the Fenton reaction, thereby accelerating the process of lipid peroxidation (Conrad et al., 2018). Our study demonstrates that, like ERASTIN, a high-glucose microenvironment can also induce ferroptosis in fibroblasts, which can be inhibited by Ferr-1. β-BA can reverse the decrease in the GSH/MDA ratio and the increase in lipid peroxidation levels caused by high glucose, thereby inhibiting ferroptosis. Furthermore, administration of β-BA in a high-glucose microenvironment can reduce cellular ferrous iron content and regulate the expression levels of ferroptosis-related genes, thus protecting the biological functions of fibroblasts. Interestingly, studies suggest that amide derivatives of β-BA can induce ferroptosis in glioma models both *in vitro* and *in vivo* (Yang Y. H. et al., 2024). This contrary result may be attributed to the dose-response curve of β-BA. According to our experimental findings, when the concentration of β-BA exceeds 80 μM, it begins to exhibit a certain degree of cytotoxicity, thereby inhibiting cell proliferation and even inducing apoptosis. At a concentration of 40 μM, β-BA promotes the proliferation and migration of fibroblasts, thereby exerting a wound healing effect. This is the first study *in vitro* to demonstrate that β-boswellic acid can effectively inhibit high-glucose-induced ferroptosis in fibroblasts.

As a core member of the STAT family, STAT3 has been demonstrated to be a crucial regulator of ferroptosis. Existing research has confirmed that STAT3 exerts its function by binding on the promoters of key molecules such as GPX4 and FTH1(Menotti et al., 2019; Ouyang et al., 2022). Interestingly, however, the regulatory effect of STAT3 on ferroptosis is associated with cell types and disease types. In an acute kidney injury model, IL - six promotes ferroptosis by reducing glutathione (GSH) in renal tubular cells through the activation of the JAK2/STAT3 axis (Dong et al., 2023). Conversely, HLF inhibits ferroptosis by promoting GSH in triple - negative breast cancer cells via the activation of the IL - 6/JAK2/STAT3 axis (Li et al., 2022). In tumors, STAT3 promotes the survival and drug resistance of cancer cells by inhibiting ferroptosis. In non-tumor diseases, STAT3 can either promote ferroptosis, exacerbating tissue damage, or inhibit ferroptosis to maintain cellular homeostasis (Xie et al., 2025). In this study, we found that under the induction of a high-glucose microenvironment, the expression level of STAT3 in HSFs was downregulated, and the administration of β-BA could partially upregulate the phosphorylation level of STAT3. Furthermore, inhibitors of STAT3 could partially abolish the improvement effect of β-BA. We discovered for the first time that β-BA can exert anti-inflammatory and anti-ferroptosis effects through the STAT3 pathway. In the future, our focus will be on upstream molecules that can target and regulate STAT3. We aim to explore the bidirectional regulatory mechanism of targeting STAT3 on ferroptosis in diabetic wounds.

Finally, we employed a diabetic rat model to validate the efficacy of β-BA. Our results demonstrated that the administration of β-BA significantly accelerated wound healing in diabetic rats. Immunohistochemical analysis provided further evidence, showing that β-BA treatment markedly reduced the level of ferroptosis in diabetic wounds (DW) and promoted collagen deposition. The advantages of using β-BA for diabetic wound treatment are evident. Firstly, β-BA has a broad spectrum of targets, as evidenced by its antioxidant stress and anti-inflammatory effects. What’s more, frankincense, its primary source, is widely cultivated in developing countries, which helps to alleviate the financial burden on these countries in managing diabetic wounds.

**FIGURE 6:**
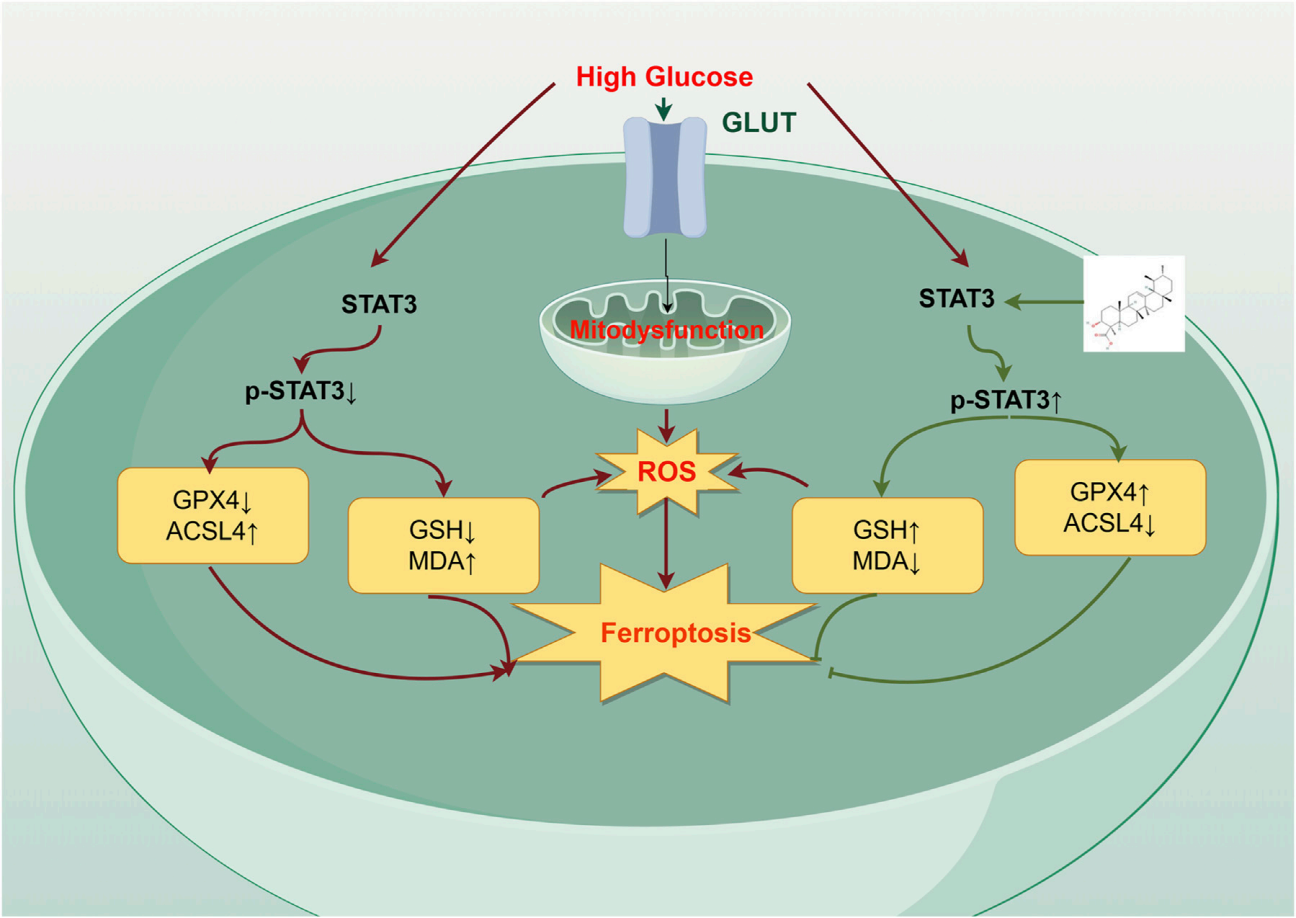
The mechanism of action of β-BA on high glucose (HG)-induced HSFs. β-BA upregulates GPX4 expression by promoting STAT3 phosphorylation levels, promoting diabetic wound healing. In addition, it alleviates HG-induced fibroblast ferroptosis by reducing the accumulation of reactive oxygen species (ROS) and mitigating cellular oxidative stress levels.

In summary, β-BA is a promising natural metabolite that promotes the healing of diabetic wounds (DW). Its therapeutic effects are attributed to inhibiting ferroptosis and modulating inflammation and oxidative stress (Figure 6). Currently, drugs containing β-BA are undergoing clinical trials, and further research is needed to promote its application in the field of diabetic wound management.

## Data Availability

The original contributions presented in the study are included in the article/supplementary material, further inquiries can be directed to the corresponding author.
